# Ultralight magnetic aerogels from Janus emulsions†

**DOI:** 10.1039/c9ra10247g

**Published:** 2020-02-20

**Authors:** Rajarshi Roy Raju, Ferenc Liebig, Bastian Klemke, Joachim Koetz

**Affiliations:** Institute of Chemistry, University of Potsdam 14476 Potsdam Germany koetz@uni-potsdam.de; Helmholtz-Zentrum Berlin für Materialien und Energie Lise Meitner Campus 14109 Berlin Germany

## Abstract

Magnetite containing aerogels were synthesized by freeze-drying olive oil/silicone oil-based Janus emulsion gels containing gelatin and sodium carboxymethylcellulose (NaCMC). The magnetite nanoparticles dispersed in olive oil are processed into the gel and remain in the macroporous aerogel after removing the oil components. The coexistence of macropores from the Janus droplets and mesopores from freeze-drying of the hydrogels in combination with the magnetic properties offer a special hierarchical pore structure, which is of relevance for smart supercapacitors, biosensors, and spilled oil sorption and separation. The morphology of the final structure was investigated in dependence on initial compositions. More hydrophobic aerogels with magnetic responsiveness were synthesized by bisacrylamide-crosslinking of the hydrogel. The crosslinked aerogels can be successfully used in magnetically responsive clean up experiments of the cationic dye methylene blue.

## Abstract 2

Magnetite containing aerogels were synthesized by freeze-drying olive oil/silicone oil-based Janus emulsion gels containing gelatin and sodium carboxymethylcellulose (NaCMC).

## Graphical abstract

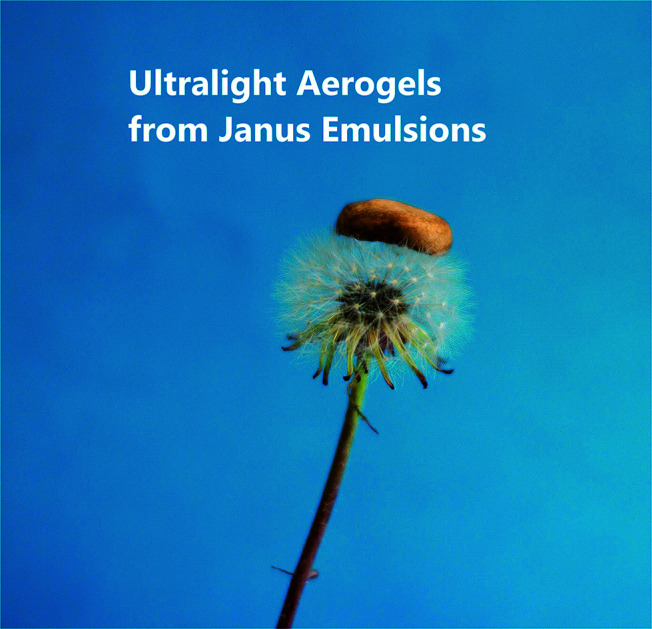

## 1. Introduction

Aerogels as a new class of porous materials were firstly introduced by Kistler in 1931 by supercritical drying of a hydrogel.^1^ However, Kistler's multistage drying process of hydrogels *via* alcogels to aerogels was complicated and not well reproducible. Therefore, aerogel related research had been neglected for a long period of time until Teichner *et al.* developed a method to synthesize silica aerogels 40 years later, by the hydrolysis of tetramethoxysilane.^2^ Afterwards, a renaissance of aerogels was initiated by advances in aerogel syntheses and improvements in the drying technology. Especially, in the last decade, a lot of research activities were focused on aerogels due to the excellent properties of the resulting ultra-low density material (<0.5 g cm^−3^) with high surface area (>500 m^2^ g^−1^) and high porosity (>80%).^3^ In the meantime, aerogels were successfully employed as insulations in buildings,^4^ aerospace,^5^ and as high-performance supercapacitors.^6,7^ In addition to these technical applications different new types of aerogels were established on the basis of nanoparticles, *e.g.*, gold,^8,9^ semiconductor quantum dots,^9,10^ iron oxide,^11^ magnetic carbon nanotubes,^12^ or graphene.^6,13^ Furthermore, nature-based aerogels on crude biomass from water melon,^14^ bagasse^15^ or cellulose fibers^16^ are available, too.

Drying can be regarded as the most critical step for successful preservation of initial morphological features of the wet gel to the final aerogel structure. Hence, many procedures are explored for drying, *e.g.*, supercritical drying, pressure drying or freeze-drying. Freeze-drying is a well-established approach to obtain so-called “cryogels”.^17–20^ A rapid freezing rate leads to smaller pore size, whereas a slow rate is accompanied by larger pore dimensions. The low-temperature process is of special relevance for biomedical applications.^17,21^ However, for some special applications, a macroporous aerogel is required to accelerate transport processes into the 3D structure. For example, biosensors related applications demanded significantly larger pore sizes to immobilize bacteria.^22^ Additionally, micro or mesopores provide a higher surface area in aerogel. Both features are important for different applications such as spilled oil sorption and separation processes.^12,23^

Therefore, a hierarchical porous structure with mesoporous and macroporous compartments is needed, which cannot be obtained by a classical freeze-drying process of a hydrogel. Emulsion-based porous systems are of interest in this context. Compartmentalized oil droplets can introduce special pore architectures and increase the interconnected macropore volume. Micro- or mesopore formation can be reinforced by adding nanoparticles and by varying the emulsifier concentration.^24^

Recently, Janus emulsions have got significant attention because of their potential application in different fields which include, detection of bacteria,^25^ fabrication of porous scaffolds,^26^ and synthesis of anisotropic particle structures by a subsequent solidification of polymerizable monomers, typically induced by stimuli.^27^ Conventionally, Janus emulsions are produced through microfluidic methods.^28^ These procedures encounter the problem of fewer throughputs and consequently, difficulties in commercialization of the processes. A pioneering work by Hasinovic *et al.* showed the first report about Janus emulsion droplets in bulk through moderate energy vibrational emulsification of two immiscible fluids (generally, oils) in a continuous phase (water, containing stabilizer).^29^ In a recent review, Ge *et al.* have presented three borderline scenarios for droplet formation through local minimization of interfacial energy in a three-phase system.^30^ The first case is the complete engulfment of one oil phase by the other, resulting in double emulsion droplets. The second is a partial engulfment of one by the other, which gives rise to the Janus emulsions, where three possible liquid interfaces are present in one single droplet. Thirdly, dispersed single oil droplets are formed, separated by the continuous phase. Depending on the extent of engulfment, five typical projections of Janus topology can be found.^31^ The droplet morphology can be effectively correlated to the interfacial tensions between the components.^32^

To stabilize Janus droplets, containing olive oil and silicone oil, the nonionic surfactant Tween 80 (ref. 29) or the amphoteric surfactant phosphatidylcholine^33^ can be used. Recently, we have shown that also copolymer,^34^ or even polyelectrolytes,^35–37^ can fulfill the equilibrium conditions for making Janus droplets. Moreover, in the presence of sodium carboxymethylcellulose (NaCMC) and gelatin polyelectrolyte complex-stabilized Janus gels are formed.^38^ In this context, micrometer-sized Janus droplets are embedded in the hydrogel matrix. Another interesting aspect of the olive oil/silicone oil-based Janus hydrogels is the possibility to incorporate magnetite nanoparticles into the oil phase.^39^

In this work, we explore a potential application of Janus emulsion-based hydrogels. The aim is to utilize the special configuration of embedded Janus droplets in a polyelectrolyte-complex (PEC) matrix, as a template for preparing magnetic responsive macroporous aerogels. In the first step, the Janus hydrogel is transformed into a cryogel network by freeze-drying. In the second step, the oil components are washed away by acetone, and the gel matrix is dried at room temperature. The resulting magnetic-responsive ultra-light material shows the typical behavior of a magnetic aerogel with hierarchical pore-architecture. As an example of a possible application, crosslinked aerogels were used in spilled oil and dye cleanup experiments.

## 2. Experimental

### 2.1 Materials

Silicone oil (viscosity 10 mPa s), olive oil, methylene blue and ethanol (≥99.5%) were obtained from Sigma-Aldrich. Iron(iii) chloride hexahydrate (FeCl_3_·6H_2_O) obtained from Fluka and iron sulfide heptahydrate (FeSO_4_·7H_2_O) from Roth were used as received. Gelatin A (Bloom number 140) was purchased from Carl Roth. Moisture contents were experimentally determined with Moisture Analyzer MA 30 from Sartorius. Carboxymethylcellulose sodium salts (NaCMC) with a degree of substitution of 0.76, synthesized in a pilot plant trial from linters cellulose, were used as provided. The specific viscosity of the NaCMC in 2 wt% cuoxam solution was *η*_spec/c_ = 0.754. Milli-Q Reference A+ water was used in all experiments. 1 wt% gelatin solution was prepared by suspending dry gelatin in water, heating up to 40 °C for one hour, followed by cooling down to room temperature under constant stirring. Aqueous NaCMC solution was obtained by mixing 1 wt% NaCMC in water and stirring overnight. Individual solutions of gelatin and NaCMC, as well as the mixture of both components at different weight ratio, were employed as aqueous emulsifiers.

### 2.2 Preparation of magnetic nanoparticles in olive oil

Fe_3_O_4_ magnetic nanoparticles were synthesized in similarity to our earlier experiments.^33^ Briefly, FeCl_3_·6H_2_O and FeSO_4_·7H_2_O in the ratio of 2 : 1 were added to deoxygenated water at 85 °C under N_2_ protection. Ammonium hydroxide is quickly added under stirring, and black colored magnetic nanoparticles (MNPs) were precipitated and washed several times with water, and a 0.02 M NaCl solution. The purified MNPs with a particle size of 13 ± 2 nm (determined by TEM measurements) were stored in a drying cabinet under vacuum. The solid magnetite nanoparticles were dispersed in olive oil by applying an ultrasonic treatment for 10 minutes. Later, the olive oil (containing MNPs) was used as given to form magnetic Janus emulsions. Unless mentioned otherwise, the concentration of magnetic nanoparticles in olive oil was 5 mg mL^−1^.

### 2.3 Preparation of Janus hydrogels

**Fig. 1:**
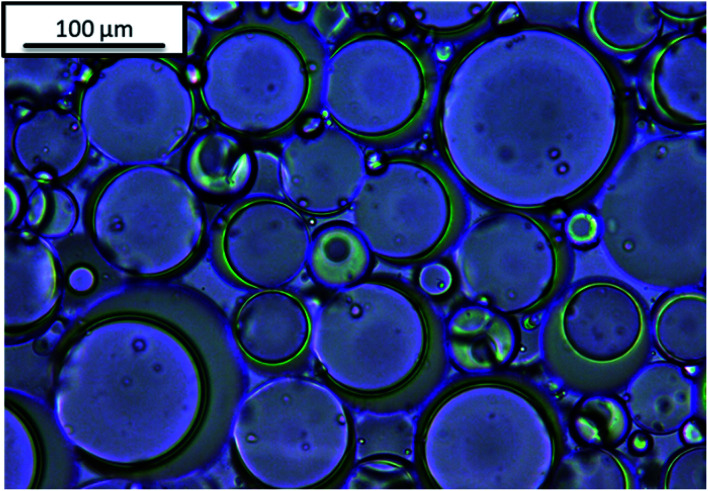
Light microscopic image of Janus emulsion gel stabilized by a complex of gelatin and NaCMC.

Janus emulsions were produced by mixing olive oil, silicone oil and the aqueous emulsifier solution in a 10 mL glass tube and homogenizing with a mini-shaker IKA (Roth) at 2500 rpm for two minutes. After one day a hydrogel is formed (compare Fig. 1), which contains Janus droplets with silicone oil as inner component and olive oil as surrounding component, in similarity to our previous experiments.^38^ Emulsions containing only one oil component, *i.e.*, olive oil or silicone oil, were prepared in comparison to the Janus emulsions. Silicone oil emulsions show phenomena of phase separation after one day, but olive oil, as well as Janus emulsions, remain long-term stable at room temperature.

**Fig. 2:**
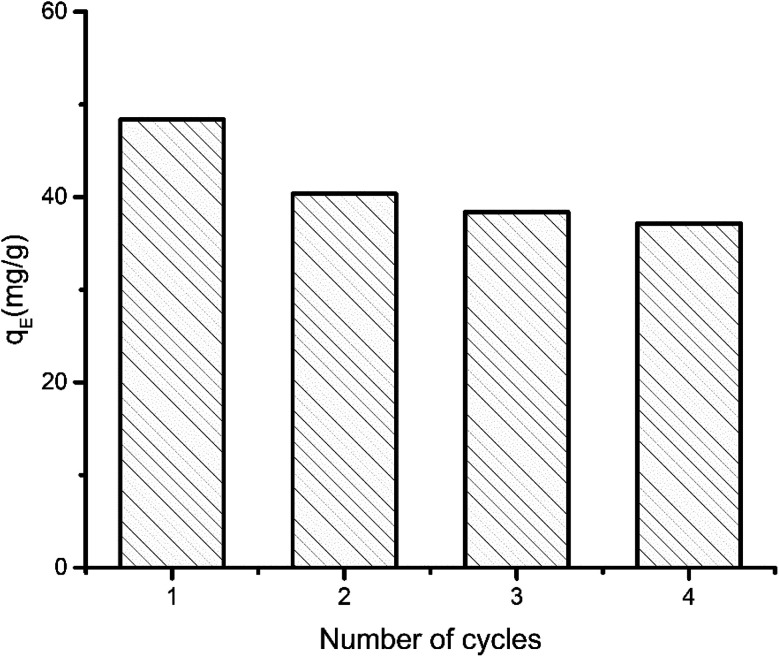
Performance of aerogel over successive adsorption–desorption cycles. Equilibrium adsorption (*q*_E_) capacity was determined from 12 hour run and initial MB concentration 50 ppm.

A similar protocol was used to form magnetic Janus hydrogels. In that case, magnetic nanoparticles were dispersed in the olive oil according to the process mentioned above. The emulsions contained 70 vol% aqueous gelatin/NaCMC solution, 15 vol% silicone oil and 15 vol% olive oil.

### 2.4 Preparation of aerogels

Emulsions were transferred to small-size petri dishes (inner diameter 5 cm and height 2 cm), pre-frozen at −18 °C for 24 hours, and subsequently nitrogen slushed before freeze-drying at <1 Pa pressure for 48 hours. A Finn-Aqua GT2 Freeze Dryer equipped with a vacuum pump was used for freeze-drying. The freeze-dried emulsions were washed by acetone to remove residual oil and allowed to dry slowly at room temperature. The morphology of the 3D porous structure of the resulting aerogel was analyzed by scanning electron microscopy (SEM).

In the case of crosslinked aerogel preparation, gelatin and NaCMC were crosslinked by using *N*,*N*′-methylenebisacrylamide (MBA) as crosslinker and potassium persulfate (K_2_S_2_O_8_, KPS) as initiator. In brief, 20 mL gelatin/NaCMC solution, mixed at 1 : 1 weight ratio, was stirred at 40 °C for 2 hours in the presence of 0.01 mol L^−1^ MBA and 0.001 mol L^−1^ KPS. The resulting aqueous solution was used as emulsifier for the preparation of aerogels, as mentioned before.

### 2.5 Adsorption capacity and reusability of aerogels

To determine the maximum adsorption capacity of the aerogel 50 mg L^−1^ of methylene blue (MB) solution was used. Briefly, 5 mg of the aerogel was dipped into 5 mL MB solution for 12 hours with gentle shaking. Afterwards, the final concentration of the dye was measured by UV-vis. The adsorbent was regenerated simply by immersing it in 0.01 M HCl and subsequently in acetone solution for few hours. 4 cycles of adsorption–desorption experiment were conducted to elucidate the reusability of the sample. Equilibrium adsorption capacity is given by,
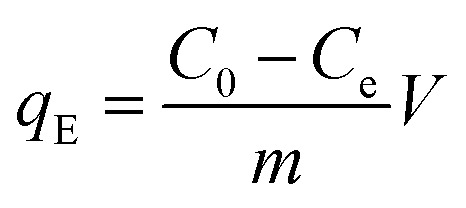
where, *q*_E_ refers to the amount of dye adsorbed by 1 g of adsorbent at equilibrium. *C*_0_ (mg L^−1^) and *C*_e_ (mg L^−1^) are initial and final equilibrium concentration of MB solution, *V* (L) is the volume of MB solution, *m* (g) is the adsorbent weight. The experiments were conducted at pH 7.5.

The maximum adsorption capacity was determined to be 48 mg g^−1^. The capacity decreased 16.16% in second cycle. Even after fourth cycle the capacity remains 76.75% of the initial adsorption capacity. The small decrease in performance can be explained by the fact that preadsorbed dye molecule remains inside the aerogel in consecutive cycles. As some of the adsorption sites in the pores are preoccupied, further adsorption of dye is marginally hindered.

### 2.6 Methods

Light microscopic images of the emulsions were obtained by the Leica DMLB microscope equipped with the Leica DFC 295 live camera. An emulsion drop was fixated on a glass slide (76 × 26 mm) overlaid with a micro cover glass (20 × 20 mm) and captured at different magnifications in direct comparison to earlier experiments.^33,35^

For scanning electron microscopy (SEM), the S-4800 from Hitachi at an acceleration voltage of 2 kV was used.

The porosity of the dried aerogels was measured by liquid displacement method using absolute ethanol according to Yang *et al.*,^40^ considering that both gelatin and NaCMC are not soluble in absolute ethanol. Open-pore volume was determined simply by the displaced amount of ethanol.

The magnetic nanoparticles (MNPs) were characterized by transmission electron microscopy using the JEOL JEM-1011 at an acceleration voltage of 80 kV. For determination of the particle size, the iTEM software was used to count the size of at least 500 nanoparticles.

Magnetization measurements of the MNPs (16.65 ± 0.05 mg, particle size: 13 ± 2 nm) and the aerogel powder (samples containing 2 mg and 4 mg MNPs, respectively) were performed using the Vibrating Sample Magnetometer option of a Physical Properties Measurement System (Quantum Design) at the Quantum Materials CoreLab (Helmholtz-Zentrum Berlin, Germany). Measurements were performed at 296 K with applied magnetic fields up to 6 × 10^4^ Oe. The error range is <5%.

For the dye related experiments, the UV-vis absorption spectra of methylene blue were recorded with a Shimadzu UV-2600 spectrophotometer at a wavelength range between 200 and 1400 nm.

## 3. Results and discussion

### 3.1 Aerogel morphology

To elucidate the significance of the Janus droplets on aerogel formation, firstly systems without oil, *i.e.*, the aqueous polyelectrolyte (PEL) solutions or their complexes were conducted. Afterwards, single oil emulsion-based aerogels were taken into consideration, followed by Janus emulsions as well as MNP containing Janus emulsions. To qualify the morphology of aerogels after freeze drying and removing the oil components, we have started to characterize the morphology of cryogels of aqueous gelatin and NaCMC solutions.

#### PEL-based aerogels

The morphology of cryogels obtained by the freeze-drying process of polyelectrolyte solutions, *i.e.*, the gelatin or NaCMC solution or the 1 : 1 mixture of both components, can be seen in Fig. S1.† In presence of gelatin (Fig. S1a†), the generation of a homogeneous structure with a lot of polygonal pores about 80 μm can be attributed to the ice crystal sublimation, in good agreement with the results obtained by Kang *et al.* for gelatin-based scaffolds.^41^ In the case of NaCMC, a disordered porous structure was observed (compare Fig. S1b†). When gelatin and NaCMC were combined (1 : 1 weight ratio) the formed pores show similarity to the gelatin-based structure. In general, gelatin-based structures are softer, and NaCMC produces relatively brittle aerogels. By combining both components, a desired mechanical property can be achieved along with the facility of retaining the ordered structure shown in Fig. S1c.†

#### Emulsion-based aerogels

Fig. S2† shows the pore morphology of aerogels obtained from different emulsions stabilized by gelatin/NaCMC (1 : 1) mixtures. The drying process can be explained into two steps. In the first step, ice crystals (formed under freezing conditions) are sublimated. However, oil droplets survive and build up the template for a 3D structure generation. In the second step, oil droplets were washed away by acetone. After a slow drying process, the final aerogel was available. When olive oil was used as the oil component a complex hierarchical porous aerogel structure was formed, where ‘longitudinal’ path was interconnected with lateral macropores (Fig. S2a†). In the presence of silicone oil (Fig. S2b†) larger unordered aerogel structures are formed. One reason for the disordered pore structure is the fact that silicone oil emulsions are not well stabilized by gelatin and NaCMC, which was immediately observed after emulsion preparation even before freezing. When olive oil or silicone oil was used as separate oil components, a large shrinkage of the overall sample takes place during the freeze-drying and the following acetone washing process (Fig. S2c†).

#### Janus emulsion-based aerogels

The problem of shrinkage could be overcome by using Janus emulsions or Janus gels containing both oil components. In that case, completely or partially engulfed Janus droplets covered by a viscous layer of gelatin and NaCMC are formed, which build up long-term stable Janus gels with thixotropic rheological properties.^38^ Different solubilization rates of the oils in acetone helps to withstand the original structure without significant shrinkage.

Gelatin itself can form Janus emulsions with olive oil and silicone oil, but the resulting emulsions are not long-term stable. On the other hand, NaCMC solely cannot stabilize Janus emulsions because of phenomena of phase separation within one hour after preparation. Gelatin is a polyampholyte, which remains positively charged below its isoelectric point (pH_iso_ = 7.2; potentiometrically determined). When gelatin is combined with NaCMC polyelectrolyte complexes can be formed, which further helps to provide resistance to coalescence of the emulsion droplets, resulting in a stable Janus gel.^38^ The initial pH of gelatin solution was 5, and 7 for the NaCMC solution, which is suitable for the complex formation.

**Fig. 3:**
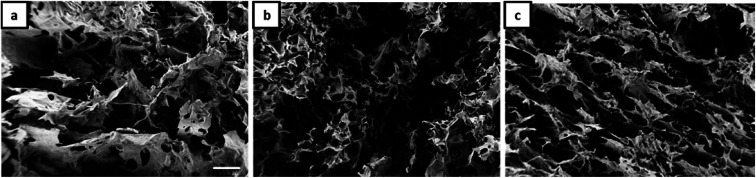
SEM micrographs (scale 100 μm) of aerogels obtained from Janus hydrogels at different weight ratios of gelatin and NaCMC: (a) 4 : 1, (b) 1 : 1, (c) 1 : 4.

The morphology of Janus-based aerogels is shown in more detail in Fig. 3 and S3† (at higher magnification) by varying the molar ratio between gelatin and NaCMC. A surplus of gelatin leads to the formation of unordered, but interconnected pores (Fig. 3a). Longitudinal, ordered porous structures can be observed with the excess of NaCMC (Fig. 3c). The pores are smaller and interconnected and build up a tube-like structure. One of the underlying reasons for different pore structures is the excess of gelatin or NaCMC. Surplus of NaCMC, *i.e.*, excess of anionic carboxylic groups, leads to the formation of a stiffer PEC-network, surrounding the emulsion droplets. In consequence, a tube-like structure (compare Fig. S3c†) is formed. However, NaCMC in the majority is detrimental to the overall stability of Janus droplets. Conversely, an excess of gelatin results in a more flexible PEC network, which could not withstand further washing processes to preserve the original structure.

**Fig. 4:**
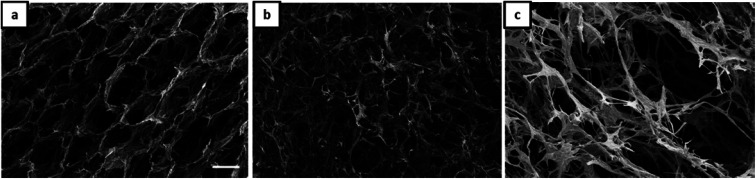
SEM micrographs (scale 100 μm) of aerogels obtained from Janus hydrogels at different weight ratios of gelatin and NaCMC: (a) 4 : 1, (b) 1 : 1, (c) 1 : 4 in the presence of magnetite nanoparticles.

As already shown by us earlier,^39^ it is possible to incorporate magnetite nanoparticles (MNPs) into the olive oil for making magnetic Janus emulsions. A representative TEM micrograph of purified nanoparticles with a mean particle size of 13 ± 2 nm is presented in Fig. S4.† When the aerogel formation process is performed in presence of magnetite nanoparticles in the olive oil, at an excess of gelatin (Fig. 4a), honeycomb-like aerogel structures of higher-order and stability can be formed. It is well-known that honeycomb-like structures are highly desired because they can provide higher mechanical strength and stability, which are relevant for many applications including thermal and acoustic insulation.^42,43^ The brown color of the aerogels indicates that MNPs are incorporated into the aerogel, not taken away during the freeze-drying, and oil removal process. Unfortunately, as the size of the MNPs is quite small (13 ± 2 nm), they could not be visualized in the aerogel network by SEM. Nevertheless, the study of the magnetization behavior of dried powdered aerogel further confirms the retainment of magnetic nanoparticles in the structure (presented later). One reason for the honeycomb-like structure is the alignment of the magnetic nanoparticles at the olive oil-polymer interface, which leads to a reinforcement of the gel structure. Interesting to note that in the absence of MNPs, the same composition leads to the formation of a more unordered structure (Fig. 3a).

Additionally, our results show that the magnetic porous structure can be tuned by varying the gelatin/NaCMC ratio (Fig. 4), which is of special relevance for applications, already shown for cellulosic material by other research groups.^44^ By increasing the NaCMC concentration an interconnected network-like aerogel structure is observed in the presence of MNPs (Fig. 4b and c). Like the scenario of Fig. 3b and c, stiffer PEC formation in the presence of higher amounts of NaCMC can induce the network formation.

**Scheme 1:**
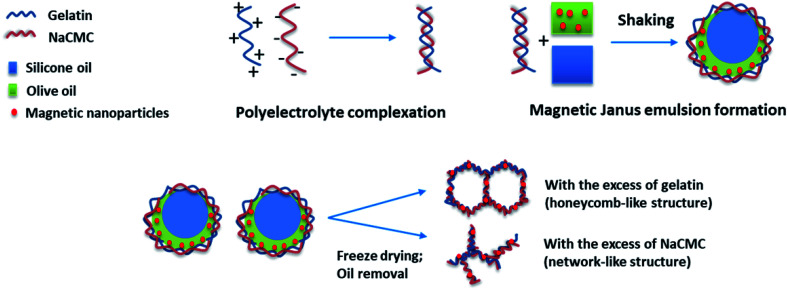
Diagram of magnetic aerogel formation *via* polyelectrolyte complex stabilized magnetic Janus emulsions.

In general one can say that the stability of the final aerogel structure can be significantly improved due to the presence of the magnetic nanoparticles (Pickering effect). The mechanism of the formation of Janus droplets stabilized by polyelectrolyte complexes and magnetic nanoparticles is illustrated in Scheme 1. In dependence on the excess component the resulting aerogels represent a honeycomb- or network-like structure with magnetic properties, which will be investigated in the next chapter.

### 3.2 Aerogel properties

**Fig. 5:**
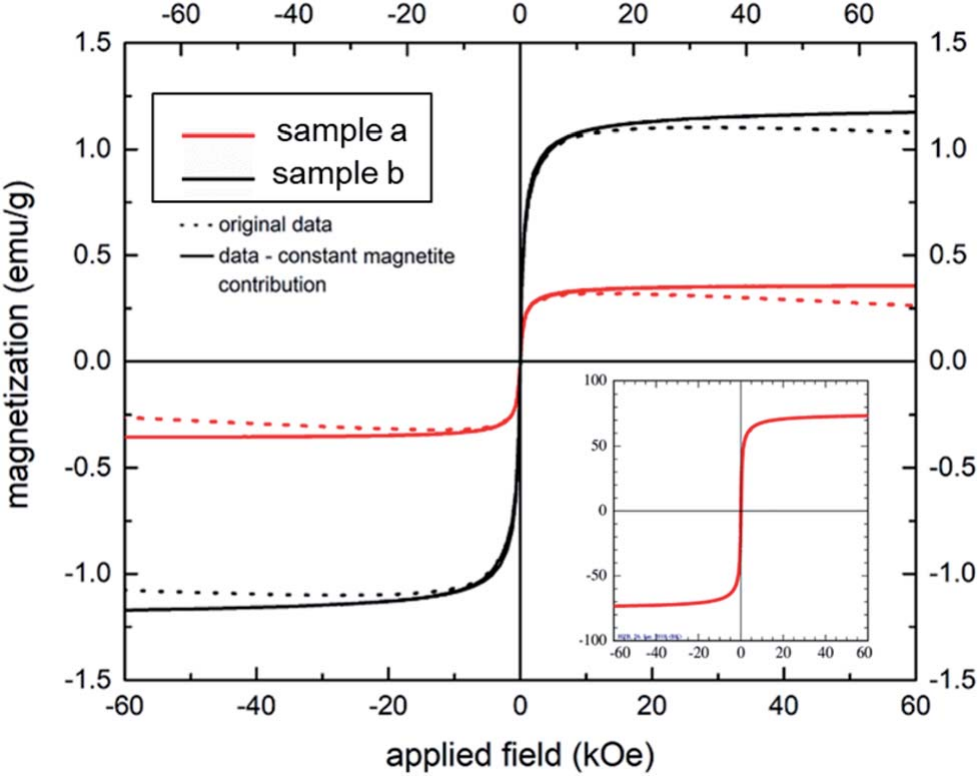
Magnetization behavior of two aerogel samples (a) and (b) (gelatin/NaCMC ratio 4 : 1) containing 2 and 4 mg of MNPs, respectively. Inset: magnetization curve of bare magnetite nanoparticles.

The S-shaped magnetization curves of two aerogel samples (a) and (b) (shown in Fig. 5) clearly demonstrate a typical superparamagnetic behavior with *M*_s_ = 0 emu g^−1^, when the applied magnetic field is zero, in full agreement with the bare MNPs (compare inset of Fig. 5). Samples (a) and (b), prepared at a gelatin/NaCMC ratio 4 : 1, contained 2 and 4 mg MNPs, respectively. The saturation magnetization of our aerogels is significantly lower in comparison to the bare MNPs (at about 70 emu g^−1^), but could be increased from 0.3 (aerogel sample (a)) to 1.2 emu g^−1^ (aerogel sample (b)) by increasing the concentration of the magnetic nanoparticles in the hydrogel. Therefore, one can conclude, that superparamagnetic properties could be successfully rendered in magnetic aerogels *via* polyelectrolyte complex stabilized magnetic Janus emulsions. Similar findings were shown by Lai *et al.*^45^ This behavior can be beneficial for tissue engineering applications.^46,47^

**Fig. 6:**
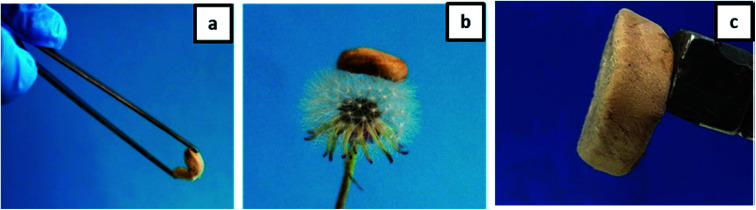
Photographs demonstrating the properties of an aerogel: (a) flexibility; (b) ultra-light weight; (c) movement by external magnet.

Furthermore, we have checked the material properties of our hydrogels. The excellent low-density properties of the material are demonstrated in Fig. 6. The high flexibility of the aerogel is shown in Fig. 6a, and the low density is demonstrated by laying the gel on the top of a flower. The whole aerogel could be moved by a household magnet as to be seen in Fig. 6c. The final porosity and density depend on the composition (see Table 1). However, the highest porosity (>89%) accompanied by a moderate density (<0.1 g cm^−3^) is observed for the honeycomb-like structure at an excess of gelatin.

**Table 1:** Porosity and density of different magnetic aerogels

Weight ratio of gelatin and NaCMC	Density (g cm^−3^)	Porosity (%)
4 : 1	0.082	89.2 ± 2.2
1 : 1	0.120	86.4 ± 3.2
1 : 4	0.057	81.5 ± 3.1

### 3.3 Crosslinked aerogels

**Fig. 7:**
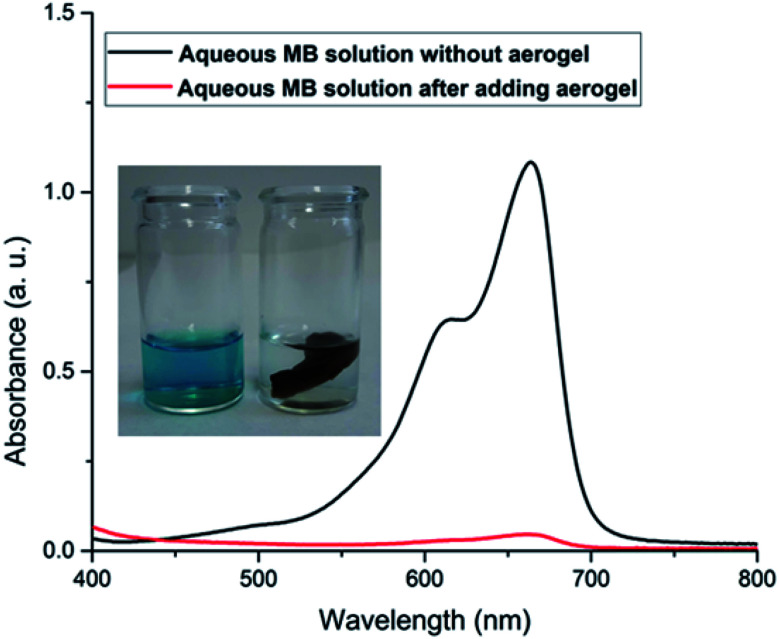
Photographs of aerogel adsorbing the cationic dye methylene blue (MB) with corresponding UV-vis spectra.

For a more conventional use of the aerogel in aqueous systems, *e.g.*, for spilled oil or dye recovery experiments,^12^ the hydrophilicity of the material should be decreased to overcome water solubility of the aerogels. It is already well-known from polyelectrolyte-based superabsorber, that this can be realized by crosslinking the polyelectrolytes.^48^ In our case, there are two options for crosslinking. On the one hand, gelatin can be crosslinked in the presence of formaldehyde,^23^ and NaCMC by adding *N*,*N*′-methylenebisacrylamide.^49^ Our results have shown that water-insoluble swollen aerogels can be produced in the presence of bisacrylamide. First experiments indicate that crosslinked aerogel composites can adsorb spilled olive oil on the water surface. The utilization of the aerogel for dye-adsorption is exemplarily shown in Fig. 7. An optical color change accompanied by a decrement of the characteristic absorbance peak of methylene blue (at 664 nm) after adding the aerogel proves the dye clean up effectiveness of the aerogel. The concentration of the cationic dye methylene blue (MB) was decreased from 3.7 ppm to 0.15 ppm due to the electrostatic adsorption of the cationic dye by the carboxylic groups of the NaCMC inside of the aerogel. The maximum adsorption capacity was determined to be 48 mg g^−1^. The reusability was checked by successive adsorption–desorption cycles. After 4 cycles the capacity remains 76.75% of the initial adsorption capacity (compare Fig. 2). The magnetic aerogels can be separated by using an external magnet. The non-suitability of the aerogel to adsorb an anionic dye (*i.e.*, acridine orange) underlines the electrostatic nature of the adsorption process.

## 4. Conclusions

Ultra-light aerogels with a honeycomb-like open pore structure can be produced by freeze-drying Janus hydrogels in the presence of gelatin, NaCMC and magnetite nanoparticles. The MNPs incorporated into the hierarchical, rigid, fiber-like 3D porous architecture of the gelatin/NaCMC network are responsible for the stability and superparamagnetic feature of the final sponge-like material after removing the oil components. The water solubility of the aerogels can be restricted by crosslinking the polyelectrolyte components with bisacrylamide. The resulting water-insoluble magnetic aerogel composites can be used for cleaning processes of polluted water by the adsorption of oil components at the oil/water interface or cationic dyes dispersed in water, followed by a magnetic separation process.

## Conflicts of interest

There are no conflict of interest.

## Supplementary Material

RA-010-C9RA10247G-s001
