# Supplementary material for: Ultralight magnetic aerogels from Janus emulsions
Source: RSC Adv. 2020 Feb 20;10(13):7492–9. doi: 10.1039/c9ra10247g (PMC9049865; doi:10.1039/c9ra10247g)
Supplement: RA-010-C9RA10247G-s001 [file RA-010-C9RA10247G-s001.pdf]

Electronic Supplementary Material (ESI) for RSC Advances.
This journal is © The Royal Society of Chemistry 2020

Supporting Information

Ultralight Magnetic Aerogels from Janus Emulsions

Rajarshi Roy Raju,[a] FerencLiebig,[a] BastianKlemke,[b] Joachim Koetz[a,*]

[a]Instituteof Chemistry, University of Potsdam, 14476 Potsdam, Germany

[b]Helmholtz-Zentrum Berlin für Materialien und Energie, Lise Meitner Campus, 14109 Berlin, Germany

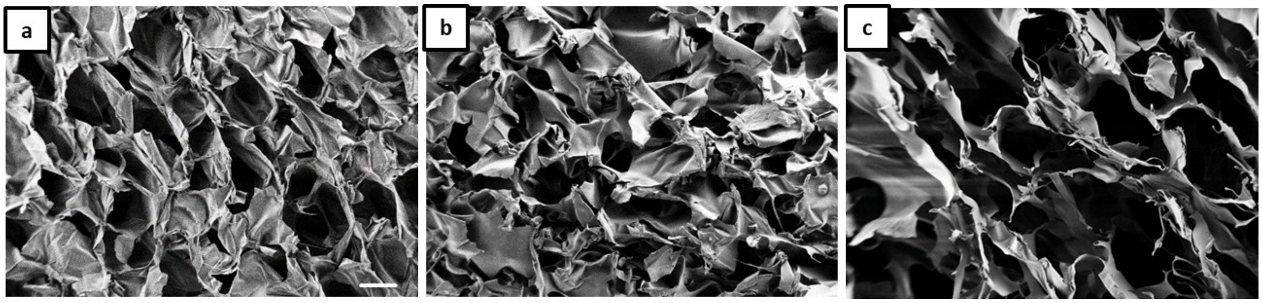

Figure S1: SEM micrographs (scale 100μm) of aerogels prepared from an aqueous solution of a) 1 wt% gelatin b) 1 wt% NaCMC c) 1wt% gelatin/NaCMC mixture.

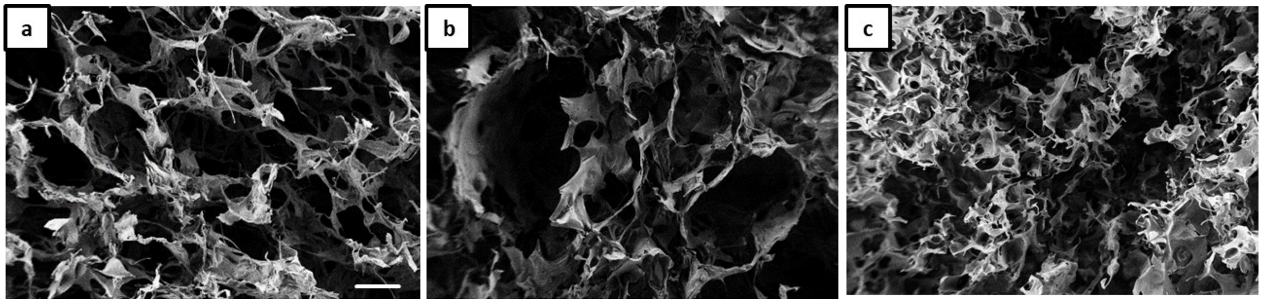

Figure S2: SEM micrographs (scale 100μm) of aerogels prepared from emulsions with a 1 wt% aqueous gelatin/NaCMC mixture and a) olive oil b) silicone oil c) mixture of olive and silicone oil

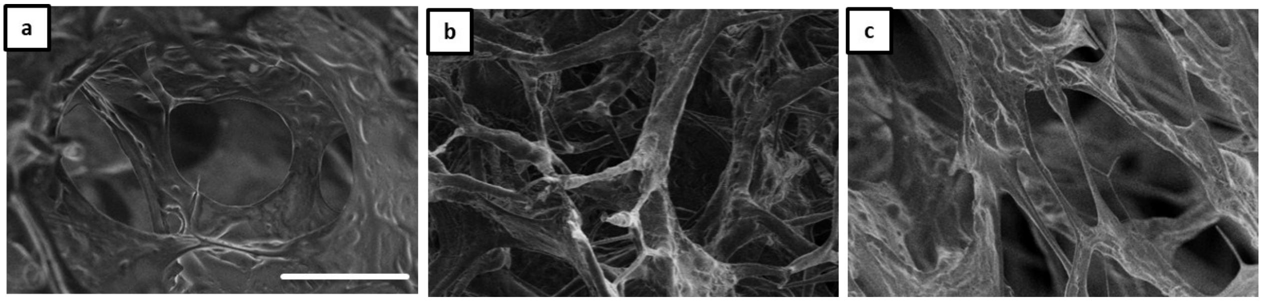

Figure S3: Enlarged SEM micrographs (scale 50μm) of magnetic Janus hydrogel-based aerogels presented in Figure 2

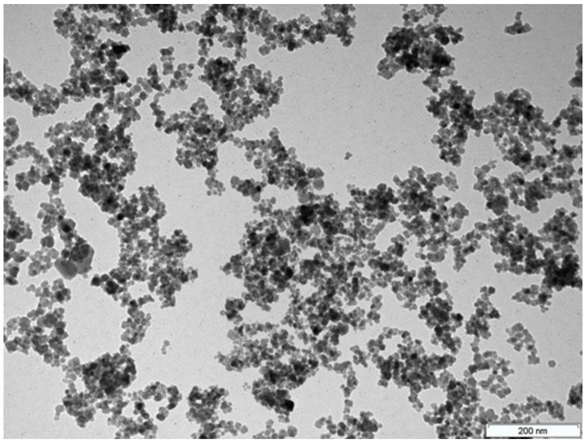

Figure S4: TEM micrograph of purified magnetite nanoparticles
